# Freshwater connectivity transforms spatially integrated signals of biodiversity

**DOI:** 10.1098/rspb.2023.0841

**Published:** 2023-09-13

**Authors:** Joanne E. Littlefair, José S. Hleap, Vince Palace, Michael D. Rennie, Michael J. Paterson, Melania E. Cristescu

**Affiliations:** ^1^ Department of Biology, McGill University, 1205 Docteur Penfield, Stewart Biology Building, Montreal, Quebec, Canada; ^2^ School of Biological and Behavioural Sciences, Queen Mary University of London, Fogg Building, Mile End Road, London, UK; ^3^ SHARCNET, University of Guelph, Guelph, Ontario, Canada; ^4^ IISD-Experimental Lakes Area, 111 Lombard Avenue Suite 325, Winnipeg, Manitoba, Canada; ^5^ Department of Biology, Lakehead University, 955 Oliver Road, Thunder Bay, Ontario, Canada

**Keywords:** habitat connectivity, fresh water, biomonitoring, environmental DNA, fish, zooplankton

## Abstract

Aquatic ecosystems offer a continuum of water flow from headwater streams to inland lakes and coastal marine systems. This spatial connectivity influences the structure, function and dynamics of aquatic communities, which are among the most threatened and degraded on the Earth. Here, we determine the spatial resolution of environmental DNA (eDNA) in dendritic freshwater networks, which we use as a model for connected metacommunities. Our intensive sampling campaign comprised over 420 eDNA samples across 21 connected lakes, allowing us to analyse detections at a variety of scales, from different habitats within a lake to entire lake networks. We found strong signals of within-lake variation in eDNA distribution reflective of typical habitat use by both fish and zooplankton. Most importantly, we also found that connecting channels between lakes resulted in an accumulation of downstream eDNA detections in lakes with a higher number of inflows, and as networks increased in length. Environmental DNA achieves biodiversity surveys in these habitats in a high-throughput, spatially integrated way. These findings have profound implications for the interpretation of eDNA detections in aquatic ecosystems in global-scale biodiversity monitoring observations.

## 1. Introduction

In fresh water, the direction and strength of water flow among habitats shapes processes of recolonization, genetic diversity, adaptation and ecological flows, and facilitates population resilience [1,2]. Within lakes or rivers, there is further partitioning according to fine-scale environmental variables such as water temperature and oxygen concentration; some organisms are adapted for life in the littoral zones of lakes while others specialize in the deeper waters of the pelagic zone. Given its importance, spatial connectivity has become a major concern in conservation when designing habitat management plans for threatened freshwater populations which are declining at a catastrophic rate [3–5]. Historically, genetic data obtained directly from inhabiting organisms have provided valuable information for inferring the biotic connectivity of freshwater habitats [6]. The non-destructive and non-invasive nature of environmental DNA (eDNA) sampling is important in contributing to species conservation goals and cultural sensitivities, as many communities reject traditional lethal survey netting [7]. However, the wider adoption of species detection based on eDNA by researchers, managers and policymakers depends heavily on our ability to accurately interpret eDNA signals, particularly when trying to distinguish organisms that currently inhabit a particular habitat, from those that inhabit nearby habitats, or organisms previously but no longer occupying the area [8]. This is particularly relevant for conservation and biodiversity projects, for example, when assessing the presence of rare or invasive species.

It is generally accepted that the complex ‘natural history' of environmental nucleic acids combined with prevailing environmental and hydrological conditions can influence the spatial or temporal resolution of species detection. While physiology, anatomy and behaviour play a role in the quantity of DNA shedding [9,10], spatial scales of detection are likely to be an interaction between the rate of initial local production of eDNA combined with subsequent dilution and transport in the environment (including vertical settling), and eventual degradation of DNA molecules [11,12]. Situations with weak transport effects (perhaps combined with high dilution) will produce local signals that rapidly dissipate in strength further from the source population: in these cases, eDNA has shown high spatial fidelity with visual or trap-based surveys [13]. For example, harbour porpoise eDNA could not be detected further than 10 m away from the animals due to dilution effects [14], and eDNA was able to distinguish vertebrate assemblages in kelp forest habitats separated by 60 m [13]. By contrast, when prevailing environmental conditions produce strong transport effects, possibly combined with high rates of initial eDNA production or low rates of dilution, eDNA signals can be transported away from the initial source population of animals, signalling regional, rather than local, biodiversity [8,15]. For example, zebra mussel eDNA was estimated to travel approximately 6 km downstream in the Gudenaa River, a 160 km lake-river catchment in Denmark [16]. To date, studies investigating eDNA transport have concentrated on downstream movement in simple lotic systems, which varies with the velocity of river flow but is likely to be a significant force in shaping nucleic acid distribution [17–19]. Other types of water movement, such as hydrological forces within lakes have been less well studied (although see [20,21]). Thus, the accurate spatial interpretation of eDNA-based surveys in aquatic networks depends on explicitly modelling the retention and flow dynamics of eDNA away from local habitats on a landscape scale.

**Figure 1.**
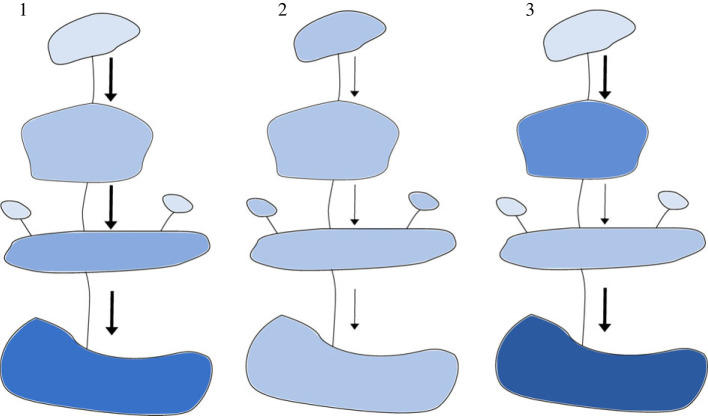
We propose a ‘spatially explicit model' in which the arrangement and the size of patches (lakes) influences the dynamics of eDNA. We assume a movement of DNA directional to the flow. For simplicity we assume that all patches can sustain equally diverse communities (as in patch dynamics perspectives [22]) and produce an equal amount of eDNA. In the figure, darker shading represents higher rates of unexpected detections with eDNA and a bolder arrow represents higher water flow between patches.

Here, we used an eDNA metabarcoding approach to analyse fish and zooplankton communities in three lake networks containing 21 Canadian boreal lakes connected only by surface flow, quantifying the spatial distribution of eDNA signals within and among lakes, as well as among networks. To best reflect the natural transport of eDNA in the environment, we focus on eDNA signatures from established fish and zooplankton populations across the trophic chain rather than experimentally introduce either caged animals or artificial sources of eDNA. We validated eDNA-based results against both current and historical population records collected since the 1970s. To investigate within-lake patchiness in eDNA signals, we examined how eDNA sampled in different zones of the lake matched known habitat use by animals (i.e. littoral, epilimnetic and deep-water). We also evaluated the frequency of downstream detection of eDNA and the influence of hydrological factors. To investigate between-lake variation in eDNA signals, we classified eDNA based on whether it matched historical and current population records as either expected or unexpected. Finally, we propose a series of spatially explicit models for the movement of nucleic acids among connected metacommunities, in which the arrangement of lakes within a network influences the dynamics of eDNA using a patch dynamics perspective [22] (figure 1).

Proposed models are as follows:

(1) *High flow networks* leading to short retention time for eDNA and high water turnover within patches; there is insufficient retention time to ensure the degradation of eDNA within the site. Prediction: eDNA reflects **regional** rather than local diversity, and unexpected detections increase with increasing lake connectivity.
(2) *Low flow networks* leading to higher retention time of eDNA within patches and slower water turnover within patches: most of the eDNA signal will degrade within the resident patch. Prediction: eDNA detections correspond to **local** diversity rather than regional diversity and the rate of unexpected detections remains relatively constant throughout the system (both within and among lakes).
(3) *Mixed networks* in which patches can have different flow regimes, some with low and some with high retention time. The shorter the retention time the more likely the flow of eDNA signal downstream. Prediction: some parts of the network retain **local** signal and more dynamic parts retain **regional** signal. Unexpected detections vary according to the local flow regime.

## 2. Material and methods

**Figure 2.**
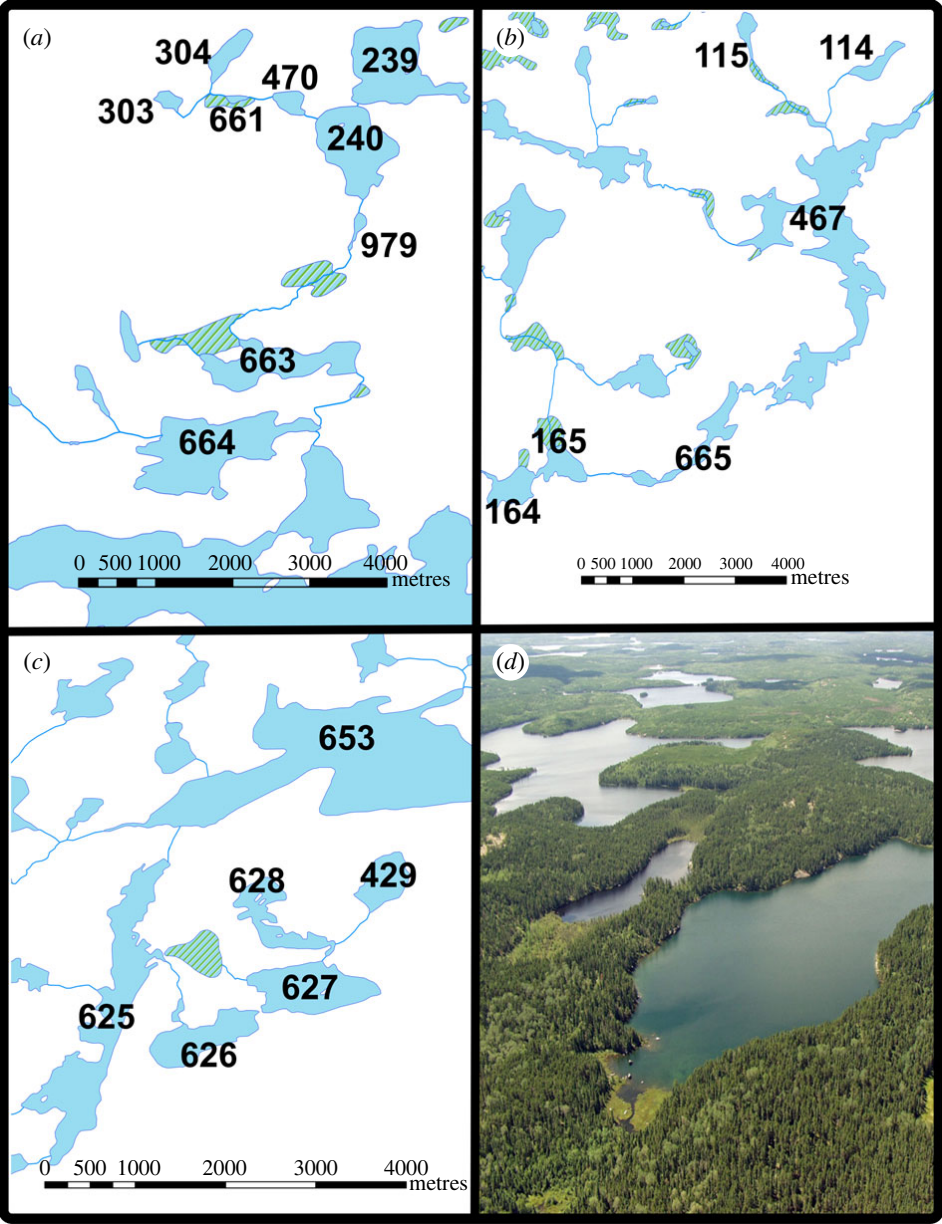
Maps of the three lake networks and aerial view of the connected lake and stream habitats of the Experimental Lakes Area, Ontario ((*a*) chain 1; (*b*) chain 2; (*c*) chain 3). Each lake at the Experimental Lakes Area has a unique identification number, which is represented on the map. An aerial photograph shows the connected lakes of chain 1 (*d*).

Sampling for eDNA was conducted from June to July 2017 at the International Institute for Sustainable Development Experimental Lakes Area (IISD-ELA), Ontario, Canada, a facility for whole-lake ecosystem experimentation and monitoring. Situated on the Canadian Shield, a geological formation dominated by granite bedrock, the region is characterized by a high density of lakes linked primarily by surface water flow with negligible groundwater flow. We collected 430 water samples from three lake chains composed of 21 lakes ranging in size from 2 to 210 hectares (figure 2; chain 1 = 9 lakes; chain 2 = 6 lakes; chain 3 = 6 lakes). Lakes in each chain were connected by streams of varying flow regimes ranging from 3.97 to 1760 m in length. We characterized lakes according to lake chain number, which measures landscape position relative to other lakes, linearly connected through surface flow [23]. We selected lake chains with the most complete historical population records of fish and zooplankton communities. Conventional monitoring and enumeration of fish and zooplankton populations in these lakes has taken place with varying levels of intensity since the 1960s. Additionally, several lakes have been monitored annually to bi-annually for fish (spring and autumn sampling) using a combination of non-lethal gillnetting and trapnetting [24,25] under Animal Use protocol no. 1464656 from Lakehead University and Licence to Collect Fish for Scientific Purposes no. 1085769 from the Ontario Ministry of Natural Resources (electronic supplementary material, table S1). Between 1968 and 2017, many of the study lakes have been sampled for zooplankton using Schindler-Patalas traps [26], nets and tube samplers [27]. We took additional zooplankton hauls in 2017 to ensure that all lakes had current species richness information, using a 30 cm diameter net with 53 µm mesh lowered to 1.5 m above the lake bed. All samples were preserved in 4% formalin after narcotization with methanol. See electronic supplementary material, note S1 for full details of conventional monitoring methods of both fish and zooplankton populations.

### (a) Environmental DNA collection and analysis

Each of the 21 lakes was sampled for eDNA using a variety of strategies. We decided the number and locations of samples based on previously published works on lakes of varying sizes [21,28]. To evaluate spatial differences across lakes, a transect of 500 ml pelagic-surface samples was taken at five evenly spaced intervals across the lake, including the deepest point of the lake (*n* = 5). To evaluate depth-specific patterns in eDNA distribution, samples along the same transect and at the same sites were taken at 1 m depth using a pole sampler (*n* = 5). In addition, deep-water samples (2 m from the sediment surface) were taken at sampling stations 3 and 4 on the transect using a van Dorn bottle (*n* = 2). We also took two samples from the shoreline of each lake, and two samples from major inflows and outflows that could be identified on each lake (electronic supplementary material, figure S1). Filtering of water samples was completed within six hours of collection onto 47 mm GF/F filters (ThermoFisher Scientific; nominal pore size = 0.7 µm). Filters were dry frozen at −20°C and transported to McGill University on dry ice for molecular analysis.

### (b) Environmental DNA molecular analysis

DNA was extracted from filters using the Qiagen Blood and Tissue kit with some modifications to the manufacturer's instructions: 370 µl buffer ATL was used in the initial incubation step, filters were incubated in ATL and proteinase K for 16 h overnight, and the DNA was eluted in 2 × 40 µl of AE buffer and stored at −80°C after elution. Extractions were treated with the OneStep PCR Inhibitor Removal Kit (Zymo Research, Irvine, CA, USA).

We created amplicon libraries with two markers; the Leray COI marker (313 bp) was used for targeting zooplankton [29] and the MiFish 12S marker (163–185 bp) was used to characterize the fish assemblages [30]. DNA was amplified in triplicate 12.5 µl reactions (technical replicates) with some changes from the original publications. For the 12S marker, we used 7.4 µl nuclease-free water (Qiagen), 1.25 µl 10X buffer (Genscript), 1 mM MgCl_2_ (ThermoFisher Scientific), 0.2 mM GeneDirex dNTPs, 0.05 mg bovine serum albumen (ThermoFisher Scientific), 0.25 mM each primer, 1 U *taq* (Genscript) and 2 µl DNA in a final volume of 12.5 µl. For the COI marker, the mastermix contained 7.875 µl nuclease-free water, 1.25 µl 10X buffer (Genscript), 1 mM MgCl_2_ (ThermoFisher Scientific), 0.1 mM GeneDirex dNTPs, 0.0125 mg bovine serum albumen (ThermoFisher Scientific), 0.2 mM each primer, 1.25 U *taq* (GenScript) and 2 µl DNA in a final volume of 12.5 µl. Thermocycling regimes included 2 min at 94°C, followed by 40 cycles of 98°C for 5 s, 50°C for 10 s and 72°C for 10 s and a final extension at 72°C for 5 min. Amplicons were run on 1% agarose gel stained with SYBR Safe DNA Gel Stain (ThermoFisher Scientific) and visualized with UV light.

Polymerase chain reaction (PCR) amplicons for each sample were combined, cleaned with AMPure beads and indexed with the Nextera DNA indexing kit for 96 samples (Illumina). A second clean-up with AMPure beads was performed, and libraries were quantified and normalized to 5 ng µl^−1^. A mock community of North American fish species was sequenced alongside our samples to evaluate the efficiency of our molecular methods and bioinformatics steps (electronic supplementary material, table S2). Equimolar amounts of DNA were combined and a total of 425 samples (419 eDNA samples, four combined blanks and two mock communities) were allocated across five sequencing lanes and sequenced with even depth per sample. Sequencing was conducted using 2 × 300 bp Illumina MiSeq at the McGill University and Génome Québec Innovation Centre, Montreal.

### (c) Contamination prevention

We structured field and laboratory work to minimize contamination between samples and lakes, as eDNA transfer between lakes was one of the fundamental research questions of our study. We sampled one lake per day and cleaned all sampling equipment thoroughly each evening. The sampling pole and van Dorn bottle were cleaned with 20% bleach between within-lake sampling points, and thoroughly cleaned with 20% bleach and soapy water at the end of each day. New gloves were used for the collection of every sample. All epilimnetic, deep-water, shoreline and inflow and outflow samples were taken in single-use whirlpak bags, which were double bagged inside a large ziplock bag. Each day, a field negative blank of autoclaved distilled water was transported into the field and filtered back in the laboratory using the same procedure as the field samples. Filtration equipment was scrubbed with hot soapy water and soaked for greater than 10 min in 30% bleach, triple rinsed with distilled water, and autoclaved before re-use. Samples were filtered in a room that had never previously been used for animal tissue or DNA work. Before work began, all floors were cleaned with floor cleaner, laboratory coats washed, and surfaces and equipment wiped down with 20% bleach. Filters were stored at −20°C in a freezer that was not used for storing animal tissue at the IISD-ELA.

DNA extraction and pre-PCR laboratory work took place in a dedicated eDNA facility at McGill University. For both DNA extraction and library preparation, only one lake was processed per day. All equipment and surfaces were wiped with 20% bleach before each use. These steps minimized potential cross-lake contamination from open tubes and multichannel pipettes. Filter tips were used at all stages of molecular work. Both negative DNA extraction and PCR controls were included for every plate by substituting with nuclease-free water (Qiagen). All filtration, extraction and PCR negative controls were amplified in triplicate. We minimized the opportunity for tag jumping by following best practice guidance to remove excess adapters and indexes with bead cleaning, store primers and indexes in small aliquots, and pool libraries just before sequencing [31].

### (d) Data analysis

We used a denoising pipeline to filter errors and cluster sequences into amplicon sequence variants (ASVs) [32]. This approach includes sorting the sequences into markers (12S and COI sequences), adapter removal, quality filtering, merging and quality control (electronic supplementary material, note S2). We assigned taxonomy to the ASVs using BLAST+ [33] with high stringency parameters (98% identity, 90% query coverage for 12S, 95% identity, 95% query coverage for COI) and used the last common ancestor algorithm in BASTA [34] to assign taxonomic identity (electronic supplementary material, note S2). We used the vegan v. 2.5–2 [35] package in R (v. 4.0.2) to compute diversity statistics and visualize species accumulation curves. We performed the following analyses to address our original objectives:

#### (i) Amplicon sequence variant abundance in relation to conventional species records

We analysed whether the number of per-sample, per-species ASV counts varied in lakes where conventional monitoring records confirmed the presence or absence of species. We used a quasi-Poisson model to account for overdispersion with ASV count number as the response variable and conventional monitoring records as a binary presence/absence explanatory variable.

#### (ii) Within-lake variation in environmental DNA distribution

To examine the contributions to within-lake patchiness in eDNA distribution, we initially analysed whether eDNA sample location in the lake (e.g. shoreline, deep-water, pelagic-surface transect, inflows and outflows) influenced the recovered eDNA community composition by performing PERMANOVA using a Bray–Curtis dissimilarity matrix with sample location as the explanatory variable. Separate models were created for fish and zooplankton data. Samples were permuted 999 times with lake identity as a strata effect.

We then conducted hypothesis testing on per-sample ASV abundances in relation to typical habitat use by each species. Fish and zooplankton specialists classified species or higher taxonomic groups according to their habitat use (fish: littoral-benthic, midwater benthic, profundal cold water, pelagic; zooplankton: littoral, profundal cold water and pelagic; electronic supplementary material, table S3). We fitted generalized mixed effects models using glmmTMB with a zero-inflated negative binomial distribution [36] to assess the interaction between species habitat use and eDNA sample location (epilimnion, deeper water, shoreline) on per-sample ASV abundances for each species. These models are appropriate for overdispersed count data. Separate models were created for fish and zooplankton. We accounted for effects that might be due to differing library sizes by including this as an offset term in the models. This approach allows us to control for library size while retaining interpretable response data (in comparison with transforming variables or rarefaction; see discussion in [37]). We allowed model intercepts to vary according to lake and species identity by including these as partially crossed random effects. We assessed the importance of the interaction between habitat preference and sample location in predicting the ASV abundances by testing significance using a likelihood ratio test (LRT) with a *χ*^2^-distribution. We used the DHARMa v. 0.4.4 package to test for overdispersion, correct handling of zero-inflated data and model assumptions [38]. Finally, we visually explored the contribution of lake size and lake state (i.e. stratified, or mixed) to the distribution of eDNA at different sample locations within the lakes.

#### (iii) Between-lake variation in species detection

We investigated our model predictions (figure 1), which describe the role of freshwater connectivity in explaining expected and unexpected species detections made with eDNA. We categorized fish and zooplankton eDNA detections as unexpected if they were not predicted by population records from current and historical surveys for the lake in question. We created statistical models with the number of species detections per sample as the response variable. Each water sample provided two datapoints based on presence/absence detection—one count of the number of expected detections and one count of the number of unexpected detections in that sample. We therefore included the filter identity as a random effect to account for the fact that each filter provided two datapoints. We included sample location (i.e. pelagic, deep-water, shoreline), lake chain number and count type (i.e. expected or unexpected detections according to conventional fishing techniques) as explanatory variables, as well as the two-way interactions between sample location and count type, and lake chain number and count type. Including these two-way interactions would investigate whether certain sample locations are predisposed to give more unexpected detections. It would also investigate whether increasing connectivity (i.e. lakes downstream in the lake network) would increase the number of unexpected eDNA detections. Initially, we also included the number of inflows to a lake in the model, but we found that this explained the same proportion of variation as lake chain number and therefore removed this term. Because species detection is likely to increase with increasing library size, we also included scaled library size as a covariate in the model to account for this. We fitted two series of models in glmmTMB using the negative binomial family (for the fish dataset) and the Poisson family (for the zooplankton dataset). We tested for overdispersion and model assumptions using the DHARMa v. 0.4.4 package to confirm that these were the best distributions to use with the respective datasets [38]. We confirmed the significance of the fixed effects terms using a likelihood ratio test with a *χ*^2^. Model reduction was performed to remove any non-significant terms, although in all cases we retained library size as a covariate, because this is a requisite part of our experimental design. We used the emmeans v. 1.7.0 package to perform *post hoc* tests to investigate the differences in how detections accumulated in lake networks for expected and unexpected detections.

#### (iv) Stream discharge and environmental DNA detections

We investigated the role of stream discharge in explaining eDNA detections in inflows that did not match detections from conventional methods. We hypothesized that streams with a greater discharge would transport more eDNA from upstream lakes, resulting in greater numbers of unexpected detections in receiving lakes. We created a subset of the dataframe with expected and unexpected detections that only included samples from these stream inflows. Discharge was measured either using weirs between lakes or by placing a current flow meter (Gurley Precision Instruments, Troy, NY, USA) at five points across the width of each stream and calculating discharge in centimetres [39]. Stream length was measured using Google satellite imagery. We then created negative binomial mixed effects models in glmmTMB for fish and zooplankton datasets as before, investigating the interaction between discharge and count type (i.e. expected and unexpected compared with conventional monitoring) on the number of detections in the samples. We also conducted some preliminary investigations to include stream length and the interaction with count type as explanatory variables, but this direction could not be pursued because of persistent issues with convergence in the statistical models. As before, we confirmed the significance of the fixed effects terms using a likelihood ratio test with a *χ*^2^-distribution and used the DHARMa package to test for model assumptions [38]. We retained library size as a covariate in all models to account for the increased likelihood of detection with larger library sizes, and included sample ID and lake ID as random effects.

## 3. Results

We detected all fish species with eDNA that were recorded by conventional techniques at the IISD Experimental Lakes Area. We also made additional detections of *Esox masquinongy* in the largest lake in our study*,* a species which is known to exist regionally. After controlling for sequences detected in blank samples and mock communities, we made 1909 detections across the dataset, with an average of 4.6 species detections per sample (electronic supplementary material, table S4). Sample accumulation curves for every lake were reflective of good sampling coverage using the 12S marker, based on our assessment of the plateaued sampling curves (electronic supplementary material, figure S2). Of detections found in the lake samples (i.e. shoreline, deep-water and pelagic-surface samples), 67% were validated by conventional current and historical fish monitoring records. Those that were predicted by conventional monitoring methods had significantly higher per-sample ASV abundances (quasi-Poisson generalized linear model (GLM), *p* < 0.001, predicted by fishing records, median: 949, interquartile range: 156–3267; not predicted by fishing records, median: 3, interquartile range: 1–122).

We made 6630 zooplankton detections with the COI dataset with an average of 31.8 ASVs per sample that could be assigned to class level or below. Sample accumulation curves indicated that sampling coverage was not as extensive as that observed for fish (electronic supplementary material, figure S2). The COI marker detected many other taxa that are not considered zooplankton and were excluded from further analysis, primarily insects. Of 264 zooplankton ASVs detected, 36 matched to Calanoida, 33 to Cyclopoida, 67 to Cladocera, 23 to Diptera and 100 to Rotifera.

### (a) Within-lake variation in environmental DNA distribution

**Figure 3.**
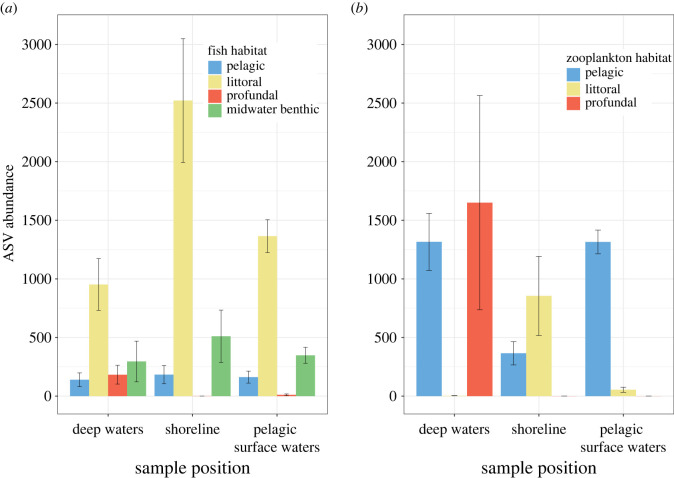
Fish eDNA ASV count is influenced by the interaction between sample location (deep-water, shoreline or pelagic-surface transect) and classification of fish habitat (littoral-benthic, midwater-benthic, profundal or pelagic; electronic supplementary material, table S3). eDNA ASV counts reflect the fish species using those habitats. Fish with a profundal habitat preference were principally found in deep-water samples, while littoral-benthic fish were predominantly detected in the shoreline samples. Zooplankton eDNA ASV count was also influenced by the interaction between the location at which the samples were collected and the habitat classification of the zooplankton (electronic supplementary material, table S3). eDNA ASV counts reflects the zooplankton species' habitat use. Zooplankton species with a profundal habitat preference were principally found in deep-water samples while littoral zooplankton were predominantly detected in the shoreline samples.

We detected different fish community compositions among different habitats within the lakes (e.g. shoreline, deep-water, pelagic-surface transect, inflows and outflows, PERMANOVA *R*^2^ = 3.69%, *p* < 0.001). Hypothesis testing showed that the interaction between fish habitat preference and eDNA sample location was a significant predictor of eDNA ASV abundances (*X* = 85.8, *p* < 0.001). In general, ASVs were most abundant when eDNA sample location matched known fish habitat preferences. In particular, ASV abundances from profundal species were highest in deep-water samples (from lake trout *Salvelinus namaycush* and slimy sculpin *Cottus cognatus*); these species were infrequently detected by shoreline or pelagic samples (figure 3*a*; electronic supplementary material, figure S3). Littoral-benthic ASVs were much more abundant in the shoreline samples (principally from either northern redbelly or fine-scale dace (*Chrosomus* spp.) and white sucker *Catostomus commersonii* in small lakes, and yellow perch *Perca flavescens* in larger lakes), and shoreline samples often detected a wide variety of species (electronic supplementary material, figure S3). Generally, the largest, stratified lakes in our study had the greatest distinctions between community compositions found in different sample types (electronic supplementary material, figure S3).

We also found strong spatial structure of zooplankton ASV abundance. The interaction between eDNA sample location and zooplankton habitat use was a significant predictor of ASV abundance (*Χ* = 147.7, *p* < 0.001). Deep-water samples had larger counts of hypolimnetic zooplankton such as *Leptodiaptomus sicilis*, but no littoral species. Hypolimnetic zooplankton ASVs were not abundant in the pelagic-surface or shoreline samples. Samples taken at the shoreline were best at detecting littoral zooplankton (in particular *Polyphemus*), which were rarely found in other locations, and samples in the pelagic-surface transect were best at detecting pelagic species such as *Diaphanosoma birgei* and *Holopedium gibberum* (figure 3*b*; electronic supplementary material, figure S4).

### (b) Between-lake variation

**Figure 4.**
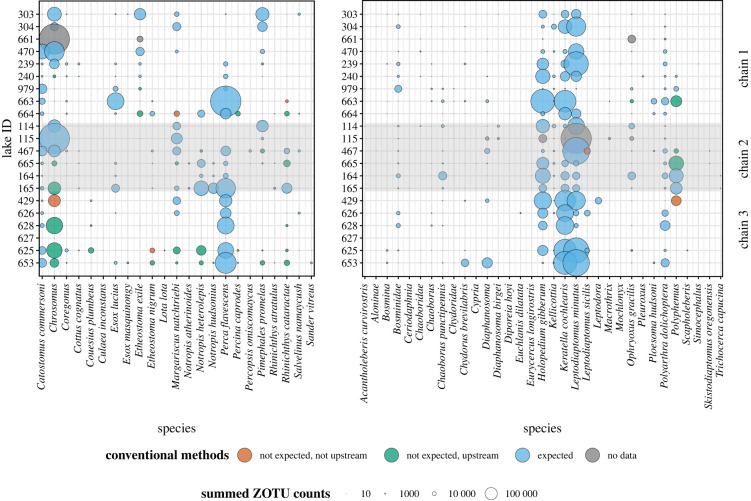
Bubble plot to show eDNA detections of each species in each lake, including all sample types. Lakes are ordered by lake network, and within each lake network they are ordered by their position within the network beginning with headwater lakes. Bubbles represent the summed ASV counts for all the samples associated with that lake, including the inflows and outflows. Bubble size is weighted by the number of ASV counts. The colour of the bubble compares eDNA detections with detections with conventional monitoring methods. Blue, expected according to conventional monitoring; green, not expected according to conventional monitoring but present in the lake immediately upstream (according to either monitoring method); orange, unexpected according to conventional methods; grey, no data on whether species is expected or unexpected because conventional monitoring is not adequate in that habitat.

**Figure 5.**
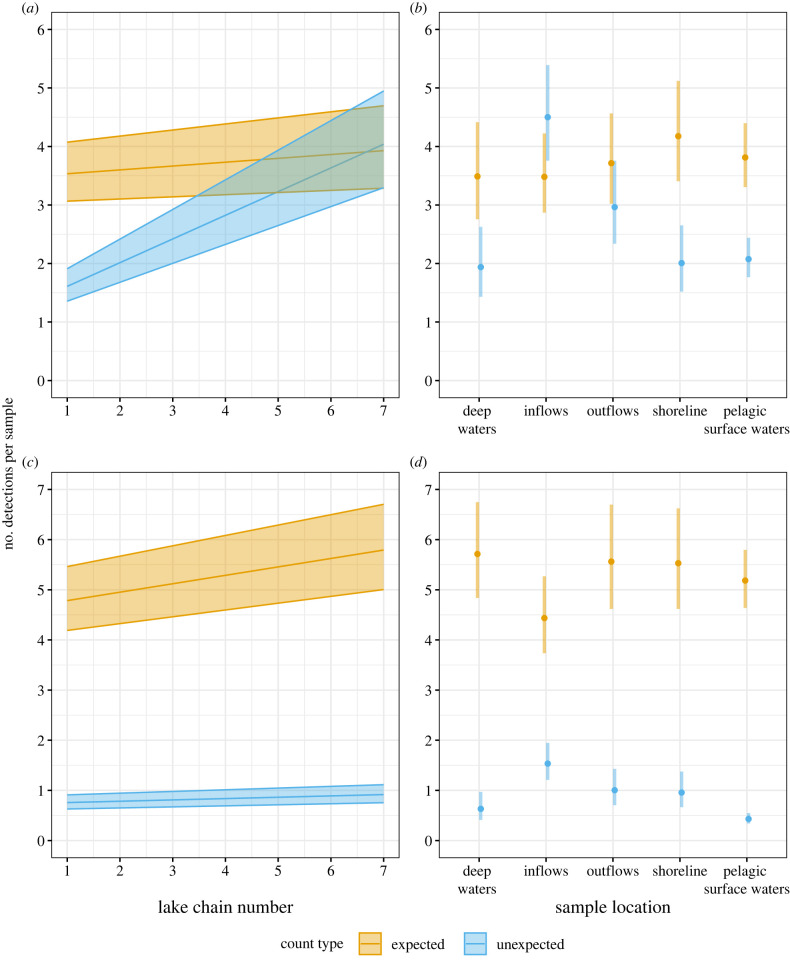
Number of per sample expected and unexpected eDNA detections as a function of lake chain number and sample location for fish (*a,b*) and zooplankton (*c,d*) datasets. Unexpected eDNA detections are those not matched by historical and current fishing and zooplankton records. Across all three networks, there is a pattern of an increase in unexpected fish species detections in downstream lakes, which is not reflected by expected detections. Plots show back-transformed model predictions from negative binomial GLMMs built in glmmTMB.

Modelling demonstrated increased numbers of unexpected eDNA detections in lakes as connectivity increased. Here we included lake chain number as a proxy for connectivity, but the same results applied to connectivity described as an increasing number of inflows (see Material and methods). For the fish dataset, there was a significant interaction between count type (i.e. expected or unexpected eDNA detections, as defined by detection compared with conventional surveys) and the position in the lake chain (figures 4 and 5; generalized linear mixed model (GLMM), LRT = 23.9, *p* < 0.001) on the number of detections per sample. While unexpected detections moderately increased in lakes further downstream in the networks, expected detections remained roughly constant throughout the networks (figure 5). These unexpected detections mostly matched species living in the lake directly upstream to the one being sampled (figure 4). In the zooplankton dataset, the interaction between count type and lake chain number was not significant. Instead, there was a significant main effect of lake chain number on all detections, with both expected and unexpected detections increasing downstream (figures 4 and 5; GLMM, LRT = 4.88, *p* = 0.027).

There was a significant interaction between sample location and count type on the number of species detected, meaning that both expected and unexpected detections were not distributed evenly throughout the freshwater networks for either fish or zooplankton eDNA (fish GLMM: LRT = 41.1, *p* < 0.001; zooplankton GLMM: LRT = 79.8, *p* < 0.001). When considering fish eDNA detections that are expected when compared with conventional methods, all sample locations detected equal species richness (figure 5*b*). This was also broadly similar to the zooplankton eDNA detections, except that there was a difference between the zooplankton deep-water and inflow locations in the *post hoc* testing, with inflows detecting fewer species matching the conventional survey (5.5 taxa in deep-water samples versus 4.1 in inflow samples, *p* = 0.024). When considering fish eDNA detections not matched by conventional methods, *post hoc* tests showed a higher number of these types of detections in inflows (mean 4.5 species) when compared with shoreline (2.0 species, *p* < 0.001), deep-water (1.9 species, *p* < 0.001), outflows (3.0 species, *p <* 0.001) and pelagic-surface (2.1 species, *p* < 0.001) samples (figure 5*b*). Zooplankton eDNA behaved similarly, with greater amounts of these mismatched detections in certain areas of the lake (figure 5*d*; GLMM, LRT = 79.8, *p* < 0.001). Specifically, higher numbers of mismatched detections were detected in the inflows (1.5 taxa) compared with the deep-water (0.61 taxa, *p* = 0.011), the outflows (0.85 taxa, *p* = 0.023), the shoreline (0.87 taxa, *p* = 0.047) and the pelagic-surface (0.40 taxa, *p* < 0.001). There were also significant differences between the outflows and pelagic-surface samples (*p* = 0.002), and between the shoreline and pelagic-surface samples (*p* = 0.002). In some instances, it was clear that non-resident eDNA was flowing from the inflow and creating a plume of DNA into the downstream lake. For example, the 159 m inflow of lake 665 contained large amounts of DNA from pearl dace (*Margariscus natchtriebi*) and longnose dace (*Rhinichthys cataractae*), both of which are residents of upstream lake 467 (chain 2, figure 5). Likewise, the 152 m inflow of lake 979 contained eDNA from non-resident Iowa darter (*Etheostoma exile*), which is a resident of upstream lake 240. The pelagic-surface sampling points closest to these inflows in lake 665 and lake 979 have a similar community composition to the water sampled at the inflow, but this non-resident signal is not apparent in further pelagic-surface sampling points. In larger lakes, the incursion of eDNA from upstream species into pelagic-surface samples was not as prominent. For example, eDNA from the zooplankton *Ophryoxus gracilis* appeared in lake 979 where it was a resident, and in the inflow of downstream lake 663, but could not be detected in the pelagic-surface transect of lake 663.

### (c) Stream discharge and environmental DNA detections

Inflow discharge did not interact with count type to influence the numbers of fish eDNA detections found in the inflow samples (GLMM, LRT = 0.0001, *p* = 0.99). Moreover, there were no significant main effects of inflow discharge (GLMM, LRT = 0.0003, *p* = 0.996) or count type (GLMM, LRT = 1.06, *p* = 0.304) on the number of eDNA detections. The zooplankton dataset displayed a different pattern, with a significant interaction between inflow discharge and count type (GLMM, LRT = 4.02, *p* = 0.045); as the inflows increased in discharge, the number of expected counts per sample increased. Unexpected counts were lower than expected counts overall, and slightly decreased as stream discharge increased.

## 4. Discussion

Our intensive sampling campaign involving 430 samples across 21 connected lakes allowed us to analyse eDNA detections at multiple scales, spanning different habitats within a lake to entire lake networks. Our results emphasize the importance of considering aquatic connectivity between metacommunities—both within lakes and flowing connections between lakes—in shaping the distribution of molecular detections. Given that 9.5% of known species occupy freshwater habitats, including one-third of the world's vertebrates, and that freshwater habitats are characterized by connectivity, our results have major implications for the application and interpretation of eDNA to characterize, survey and monitor aquatic communities [4].

Lakes with greater connectivity (i.e. those further downstream or with a greater number of inflows) had a greater number of detections of fish eDNA that did not match conventional methods. This analysis only included data from samples collected within the lakes themselves (i.e. not samples collected in inflow and outflow streams or surrounding ponds), thus precluding the contribution of a greater number of inflow samples to the accumulation of additional detections. Occasionally, the unexpected eDNA signal was very strong, being found in the majority of samples from a lake. This suggests that eDNA is detecting animals that have not been caught with conventional techniques. For example, salmonid signals from lake 303 point to either residual sedimentary DNA or a few live individuals living in the lakes after an old species introduction experiment was conducted in 2011 [40], despite the fact that the species in question was thought to be extirpated after the end of the experiment. There was a similar pattern with the eDNA of the two *Chrosomus* species which was found with large read counts in samples in lake 429. Because this is a headwater lake, this cannot be explained by downstream flow of eDNA; however, the signal was strongest in a wetland area at the outflow of lake 429 which would not usually be sampled with conventional fishing techniques. The eDNA signal appeared to dissipate with increasing distance from this area, so it is possible that *Chrosomus* are occupying part of the wetland and outflow which is not sampled with conventional techniques, and this signal is dispersing via mixing back into the epilimnion of this small and shallow lake. Despite these few occurrences, long-term ecological research sites are some of the best places in the world to perform this kind of comparison between molecular and conventional fishing techniques, because of the comprehensive sampling effort and extensive species databases covering many lakes. It is likely, therefore, that at least part of unexpected eDNA detection can be explained by DNA molecules flowing from upstream lakes into the inflows and mixing with the downstream epilimnion. While we might expect downstream lakes to have a greater species richness generally due to increasing lake size, downstream lakes were not consistently the largest in our study. Moreover, the rate of increase in unexpected detections was much higher than the moderate increase in expected detections further down the networks. Previously, invertebrate eDNA has been shown to travel between 9 and 12 km downstream from a river flowing downstream from a lake [17]. Other studies have also shown the transport and accumulation of eDNA on the scale of several kilometres [8,15,41–43]. With regard to our model scenarios, this points to a model similar to scenario 1—the propensity for the downstream accumulation of DNA, in which high flow and low retention times do not allow for the complete degradation of eDNA within a lake and thus molecular signal accumulates with greater freshwater connectivity.

Inflows and outflows create fine-scale physical, chemical and biological heterogeneity across lakes [44]. We found heterogeneous eDNA signals in both the inflows and outflows of lakes which differed from samples taken within the lakes. Species richness detected in inflows was high compared with samples from other locations. Moreover, there were frequent ‘unexpected' detections in inflow samples, albeit generally at low read counts, which did not match the composition of the receiving lakes according to decades of conventional monitoring [45]. Sometimes small littoral fish and zooplankton live in streams or close to the outflow of the upstream lake, which resulted in the frequent transport of eDNA of these species in our study. This may partially explain the patterns of downstream detections from *Chrosomus* species, which was the most frequent type of eDNA transported from upstream lakes. However, we also found eDNA originating from other fish species that would not normally dwell in streams. In some cases, this DNA was only detected in the inflow, but in the case of pearl dace eDNA from lake 467 flowing into lake 665, and Iowa darter eDNA flowing from lake 240 into lake 979, it was also detected within the pelagic-surface samples nearest the inflow (but not in further pelagic sampling points). This plume of non-resident eDNA could be facilitated by the fact that these receiving lakes are reasonably narrow ‘channel'-shaped lakes coupled with a proportionally large inflow. It is possible that morphometry combined with the degree of connectivity is an understudied determinant of the level of incursion from residual molecular signal. As biologists increasingly work on the restoration of ecological communities in rivers that have a history of channelization, this will be a useful consideration when considering biomonitoring of these habitats with eDNA. Stream substrate has been identified as another contributing factor for the retention and release of eDNA, with larger grain sizes flushing eDNA more rapidly from the system [19]. The streams at IISD-ELA are composed of small gravel sizes coupled with some larger rocks typical of Canadian Shield geology, with little fine sediment or silt. We might therefore expect moderate retention of eDNA in the connecting streams in this study, compared with habitats with larger cobbles or artificial bank reinforcement. Studies using the release of artificial DNA point to the short-lived post-release detection of DNA in streams [19], but in our view, it is essential to observe eDNA release from established populations in natural systems, as these will capture the long-term balance between eDNA shedding, dilution, transport, decay, sedimentation and resuspension.

Although the streams at IISD-ELA are relatively small, they are also short in length, and can therefore contribute to downstream eDNA signals before the molecules degrade. Intuitively, we might expect increased stream discharge to carry eDNA further downstream [46]. However, the relationship is more complex than previously thought, as increased water volume has been shown to have a moderately diluting effect on eDNA copy number [47,48]. Larger rivers will also have high flow but limited lateral movement of eDNA across the channel, meaning that detection of eDNA downstream may not always be consistent [49], whereas the streams in our study were small and well mixed. Flow regime might also act indirectly on eDNA detectability by affecting other abiotic factors such as the levels of inhibitors in the water, degree of particle settling or resuspension of particles from the streambed [19,50]. Other studies demonstrate a seasonal effect of eDNA transport in inflows and accumulation in downstream habitats [47,51]. At the time of our sampling, some inflows were slow flowing as it was the height of summer. While some flash rain events did occur during our six-week sampling period, stream flow is typically driven by spring snowmelt and early summer rain in this region. Simply measuring stream discharge at single time points may be less likely to reflect the potential transport of eDNA among lake ecosystems, compared with a more integrated picture of ongoing eDNA accumulation in downstream lakes which could be achieved by repeated measurements over time. The complexity of interacting factors combined with the intermittent nature of these streams may explain the lack of a simple relationship between stream discharge or length and eDNA transport that is consistent across fish and zooplankton eDNA.

We have demonstrated robust evidence for the spatial partitioning of DNA signals within a lake. Largely, the habitat preferences of fish and zooplankton defined the community composition of the eDNA signals found in those sample locations. Strikingly, the thermocline seems to be an important factor in restricting eDNA flow to surface waters, as hypolimnetic species are almost exclusively detected in profundal cold water. During the summer months, some zooplankton e.g. *Leptodiaptomus sicilis* and fish species like lake trout (*Salvelinus namaycush*) and slimy sculpin (*Cottus cognatus*) are isolated to profundal cold water below the thermocline due to their oxythermal habitat requirements, and we found that their eDNA was almost exclusively detected in those respective environments. This is a seasonal pattern driven by stratification of lakes, which isolates both cold water species and their eDNA to the bottom of lakes during the warmer months [20,52,53], resulting in little hydrologic connectivity between the epilimnion and hypolimnion. The limited activity of these species during the warmer months may also play a role in the low volume of DNA shed through gametes, cells and faeces [9,10,54]. Studies using radioactively labelled water added to similar sized lakes showed that there is very limited diffusive exchange across the thermocline and concluded that the thermocline acts as a barrier inhibiting the downward transfer of turbulent mixing energy [55,56]. This could explain the infrequent detection of DNA belonging to cold water species in any other parts of the lake. Some of our study lakes were too shallow to stratify. Our sampling design still incorporated a water sample from the bottom of these small, shallow lakes, but in these cases the detected community composition was more similar to samples from the shoreline and pelagic-surface waters (electronic supplementary material, figures S3 and S4). In large, stratified lakes, however, the detected community composition differed greatly between deep-water samples and shoreline/pelagic-surface samples.

eDNA from littoral fish species was spatially structured between the shoreline and pelagic samples, with samples at the shoreline containing more littoral fish sequences from species such as fathead minnows (*Pimephalas promelas*), yellow perch (*Perca flavescens*) and blacknose shiner (*Notropis heterolepis*). This pattern was even more pronounced with littoral zooplankton, which were very rarely detected in pelagic or deep-water samples. In particular, *Polyphemus pediculus*, a predatory littoral cladoceran, had very high sequence numbers in shoreline samples. We found the separation of distinct littoral and pelagic communities surprising, because radio-tracer experiments in IISD-ELA lakes have shown that the epilimnion is fully mixed within 1 day of tritiated water injection [56], due to wind stress on the lake surface. However, the heterogeneous eDNA signal originates from the larger lakes in our study, where distinct community compositions were detected between shoreline and pelagic epilimnion samples, reflecting the habitat preferences of these species (electronic supplementary material, figure S4). These larger lakes probably support unique littoral and pelagic fish and zooplankton communities, as well as presenting longer times for eDNA signals to mix across the epilimnion. Other eDNA studies from single lakes have hinted at this finding; for example, small littoral fish were found to have greater relative sequence abundances in shoreline samples compared with samples from the centre of large lakes (1480 ha [53]; 122 and 4343 ha [57]). The greater separation of signal from littoral and pelagic zooplankton communities when compared with littoral and pelagic fish communities might be due to the fact that fish have a larger body size or a different eDNA shedding rate compared with zooplankton, or possibly they are able to range more widely into the centre of the lakes beyond the littoral habitat. eDNA shedding rates, influenced by animal physiology, behaviour and metabolism, will interact with spatial and landscape factors in order to create the final eDNA signal [9,10,54].

Within lakes, heterogeneity in species detection with eDNA is shaped by heterogeneity in habitat and thermal structure. Especially in larger lakes, eDNA signals had spatial structure that reflected the habitat preferences of animals. There is also clear evidence that eDNA can reflect upstream communities of organisms when a high degree of ecosystem connectivity is present. Using a landscape perspective of freshwater ecology, lakes are explicitly viewed as connected to each other and their catchment area. eDNA does not accumulate homogeneously downstream, but both landscape factors (i.e. the position of the lake relative to others in the network) as well as individual lake-specific factors such as morphometry influence the degree and mobility of eDNA signal. We have highlighted how motion in water, which is a fundamental process in freshwater systems, will shape detectable eDNA signals and therefore biomonitoring sampling designs.

## Data Availability

Data are available from the Dryad Digital Repository: https://doi.org/10.5061/dryad.zs7h44jd0 [58]. Additional information is provided in electronic supplementary material [59].
